# Comparisons of Language Characteristics Using the Western Aphasia Battery Among Patients with Word‐Finding Difficulty: A Pilot Study

**DOI:** 10.1002/alz70857_104166

**Published:** 2025-12-25

**Authors:** Juyoun Lee

**Affiliations:** ^1^ Chungnam National University Hospital, Daejeon, Korea, Republic of (South)

## Abstract

**Background:**

Word‐finding difficulty is a common complaint among aging individuals. It could be presented in normal aging, Alzheimer's disease (AD), or primary progressive aphasia (PPA), which may initially seem similar but have very different prognoses. This study aimed to explore language characteristics in patients with word‐finding difficulty and compare linguistic differences between their clinical diagnoses.

**Methods:**

This study enrolled 18 patients who complained of word‐finding difficulty. Language profiles were assessed using the Aphasia quotient (AQ), a part of the Korean version of the Western‐Aphasia Battery (K‐WAB). Cognitive functions were evaluated through the Mini‐Mental Status Examination (MMSE), the Clinical Dementia Rating Scale (CDR), and the Seoul Neuropsychological Screening test, also including the Boston Naming test (BNT), comprehension, repetition, and category/phonemic fluency. Participants were divided into AD, non‐fluent variant PPA (nfvPPA), and other PPA groups, and their characteristics were compared.

**Results:**

AD patients (27.8%), confirmed by amyloid positron emission tomography, showed the highest AQ scores but showed significant deficits in controlled oral word association tasks (COWAT), and BNT. nfvPPA patients (27.8%) showed the lowest AQ scores and difficulty with Yes/No questions and command in the comprehension part. The other PPA group (44.4%), which was not obvious nfvPPA and lacked confirmed neuropathology, showed mixed language characteristics. Impairment in COWAT was consistent in all patients with word‐finding difficulty, even in mild cognitive impairment. Unlike AD patients, the nfvPPA and PPA groups showed worse frontal/executive dysfunction and neurologic deficits such as gait difficulty, dysphagia, dysarthria, and rigidity.

**Conclusion:**

Among language tests, COWAT, commands, and Yes/No questions differed among patients with word‐finding difficulty. However, given the small sample size, these findings are preliminary. Further research with larger populations is needed to determine whether the identified factors can serve as reliable markers for differential diagnosis.

